# Evaluation of the Association between the AC3 Genetic Polymorphisms and Obesity in a Chinese Han Population

**DOI:** 10.1371/journal.pone.0013851

**Published:** 2010-11-04

**Authors:** Hairu Wang, Ming Wu, Weiguang Zhu, Jin Shen, Xiaoming Shi, Jie Yang, Qihui Zhao, Chuan Ni, Yaochu Xu, Hongbing Shen, Chong Shen, Harvest F. Gu

**Affiliations:** 1 Department of Epidemiology and Biostatistics, School of Public Health, Nanjing Medical University, Nanjing, People's Republic of China; 2 Center for Disease Control of Jiangsu Province, Nanjing, People's Republic of China; 3 Center for Disease Control of Suqian City, Suqian, People's Republic of China; 4 Division of Chronic Disease Control and Community Health, Chinese Center for Disease Control and Prevention, Beijing, People's Republic of China; 5 Department of Molecular Medicine and Surgery, Rolf Luft Center for Diabetes Research, Karolinska Institutet, Karolinska University Hospital (Solna), Stockholm, Sweden; Mayo Clinic College of Medicine, United States of America

## Abstract

**Background:**

AC3 is one of adenylyl cyclase isoforms involved in cAMP and insulin signaling pathway. Recent reports have demonstrated that the AC3 genetic polymorphisms are associated with obesity in a Swedish population. AC3 knock out mice exhibit obese when they age. These findings suggest that AC3 plays an important role in the regulation of body weight.

**Methodology/Principal Findings:**

In the present study, we evaluated the association between the AC3 genetic polymorphisms and obesity in a Han Chinese population. A total of 2580 adults, including 1490 lean (BMI = 18.5–23.9), 677 overweight (BMI 24.0–27.9) and 413 obese (BMI ≥28.0) subjects were genotyped for 5 TagSNPs in the AC3 gene. Single maker association analyses indicated that SNP rs753529 was significantly associated with BMI in obese subjects (P = 0.022, OR = 0.775 95%CI = 0.623–0.963), but not in overweight subjects (P = 0.818). Multiple maker association analyses showed that the haplotype (G-G-G) constructed with SNPs rs1127568, rs7604576 and rs753529 was significantly associated with obesity (P = 0.029). Further genotyping of SNP rs753529 in 816 children, including 361 overweight subjects (BMI>P_80_) and 455 controls (BMI = P_20–50_) were performed, and no significant association with BMI was found. All tests were adjusted for age, sex, physical activity index, household income and/or diet expenses.

**Conclusions:**

The present study provides replication evidence that the AC3 genetic polymorphisms are associated with decreased risk of obesity among adults but not in children in a Chinese Han population. The data also suggest that the AC3 genetic effects on BMI may have interaction with the factors related to ageing and environment.

## Introduction

Obesity is a major public health problem in both developed and developing countries because it is causally related to a wide spectrum of chronic diseases including type 2 diabetes, cardiovascular diseases and cancer [1], [2]. In China, more than one-third of adults are overweight or obese which defined as BMI ≥24 kg/m^2^ and 28 kg/m^2^ respectively and that definition considered a lower BMI cut-off point should be recommended in the prediction of risk of T2DM and CVD for Chinese people [3]. Body weight control in children has become a crucial problem to be taken into the appropriate educational and intervention programs [4]–[6]. Obesity is a complex disease, which is influenced by genetic and environmental factors [7], [8]. Identification of the susceptibility and resistance genes in this disease will provide useful information for better understanding its patho-mechanisms and may subsequently lead to development of novel therapeutic approach.

Adenylyl cyclases (ACs or ADCYs) are enzymes that catalyze the synthesis of cyclic 3′5′-AMP (cAMP) from ATP. There are 10 closely related isoforms including ACs 1-9 and AC activating polypeptide 1 (ADCYAP1) that have been cloned and characterized in mammals [9]. AC3 (OMIM: 600291) is the third member in the AC family, and the aliases for AC3 gene are ATP pyrophosphate-lyase 3 and adenylate cyclases III. This gene is located in chromosome 2p23.3. Recently, a genetic association study has indicated that the AC3 genetic polymorphisms are associated with decreased risk of body mass index (BMI) among the subjects with obesity and type 2 diabetes in a Swedish population [10]. Furthermore, AC3 knock out mice exhibit obese when they age mainly due to low locomoter activity, hyperphagia and leptin insensitivity [11]. These two studies from genetic and functional analyses provide evidence suggesting that AC3 is a novel gene in the regulation of body weight [12]. However, whether the AC3 genetic polymorphisms are associated with obesity in Chinese population is unknown. To address this question, in the present study, we have carried out a genetic study of the AC3 gene in a Chinese Han population, including 2580 adults and 816 children.

## Methods

### Subjects

Totally, 2,710 adult subjects with age from 18 to 62 years old were recruited from a rural population of 14,469 subjects in two townships near 10 kilometers apart in eastern Jiangsu by the epidemiological stratification sampling approach. According to the criteria of overweight/obesity from working group on obesity in China (WGOC, 6), 413 subjects with body mass index (BMI)≥28 kg/m^2^ were defined as obesity, while 677 subjects with BMI from 24 to 27.9 kg/m^2^ were defined as overweight. Additional 1490 subjects with BMI from 18.5 to 23.9 kg/m^2^ were taken as the group of lean subjects. 130 subjects with BMI <18.5 kgm^2^ were excluded from the study. The ethics committee of Nanjing Medical University has approved the research protocol. During epidemiological interviews, written informed consent was given to all subjects and trained research staff administered a standard questionnaire to obtain information on demographic characteristics including age, gender, nation, education, occupation, household income and physical activity. At the same time, physical examination, including body height and weight measurements was conducted and repeated twice. The subjects with secondary obesity, coronary heart disease and chronic kidney disease were excluded from the study. We used a self-reported 24-hour physical activity instrument, which was simplified version of international physical activity questionnaire (IPAQ) [13], [14]. Physical activity index (PAI) was calculated as the product score of hours and MET (metabolizable energy estimate) of physical activity according to the averaging MET (hd^−1^) of 24 hour activities, including sleeping (MET), watching TV or sitting (1.1 MET), light activity (1.5 MET), moderate activity (4 MET), and vigorous activity (8 MET). Household income status was evaluated as average income (Chinese Yuan CNY/person/year), which was calculated as whole year household income and divided by the family members.

Additionally, 816 children subjects at the age of 5–15 years old were recruited from 2,373 children in another district. Of them, 361 subjects with BMI over 80th percentile (P_80_) per age group (1 year) were taken as overweight cases and 455 subjects with BMI from 20th to 50th percentile (P_20–50_) per age group (1 year) were included as the controls. All families of children accepted informed consent and completed the questionnaire to obtain information on demographic characteristics including age, gender, nation, grade, transportation and distance to school, food behavior and expenses. All the children were measured body height and weight. Since the frequency and strength of PAI of children were different from adults, PAI was then calculated as the product score of hours and averaging METs of physical activity after leaving school, including watching TV or sitting (1.1 METs), walking (1.5 METs), cycling (4.0 METs) and sport (8.0 METs).

Characteristics of all adult and children subjects included in the present study were represented in Table 1.

**Table 1:** Characteristics of adult and children subjects according to BMI.

Variable	Adult subjects			Children subjects	
	BMI = 18.5–23.9(n = 1490)	BMI = 24–27.9(n = 677)	BMI≥28(n = 413)	BMI = P_25–50_ (n = 455)	BMI>P_80_(n = 361)
Age (years)	50.8±6.6	51.4±6.4	50.5±6.8	10.1±2.9	10.0±3.0
Sex (male %)	36.3	35.8	27.9*	52.7	56.4
BMI	21.5±1.5	25.7±1.2*	30.6±2.2*	16.43±1.53	21.27±3.85*
PAI	64.2±21.1	59.0±22.4*	58.2±21.9*	29.94±0.80	29.94±0.81
Household income (CNY/year)	5633±3758	6378±4979*	6072±3995*		
Diet expending (CNY/day)				9.63±5.85	11.31±6.38*

Data were means ± SD; BMI  =  body mass index; CNY  =  Chinese Yuan; PAI  =  physical activity index; Comparison tests with one-way ANOVA (LSD) in adults and with student t test in children were performed;

*P-value <0.05.

### SNP selection

The AC3 gene spans 113,017 bps and consists of 21 exons. We searched for tagger single nucleotide polymorphisms (TagSNPs) from the data of Chinese Han population in Beijing, China (CHB) in HapMap (HapMap Data Rel 24/phase II Nov08, on NCBI B36 assembly, dbSNP b126). All TagSNPs were selected with minor allele frequency (MAF) ≥0.05 and r^2^≥0.8 according to the linkage disequilibrium (LD) values. Two SNPs, rs2033655 and rs1968482 have been recently reported to associate with obesity in a Swedish population [10], and these two polymorphisms were included in the present study. All selected SNPs were validated with 96 DNA samples in test experiments. SNP rs2033655, which is located in the promoter/regulatory region of the AC3 gene, had less 0.05% MAF in the studied population. And so, another functional SNP of rs11676272 which was linked to rs2033655 (r^2^ = 0.862) was selected as candidate locus of AC3 gene. Finally, 5 TagSNPs were included in the genotyping experiments. Information of SNP ID, type and location are summarized in Table 2.

**Table 2:** MAFs of the AC3 genetic polymorphisms in a Chinese Han population.

SNP ID	SNP type	SNP location	Lean subjects (BMI 18.5–23.9)	Over-weight subjects (BMI 24.0–27.9)	Obese subjects (BMI≥28.0)
rs11676272	C/T	Exon 1 Ser107Pro	T 0.423	0.433	0.400
rs1968482	A/G	Intron 2	G 0.384	0.413	0.361
rs753529	A/G	Intron 11	G 0.356	0.369	0.322
rs7604576	A/G	Intron 15	G 0.332	0.350	0.314
rs1127568	G/A	Exon 17 Ser957Ser	A 0.130	0.129	0.130

MAF  =  minor allele frequency.

### SNP genotyping

DNA was extracted according to a standard phenol-chloroform method [15]. Genotyping experiments were performed with the protocol of polymerase chain reaction and restriction fragment length polymorphism (PCR-RFLP). Information on primers, PCR-RFLP conditions is available, upon request. The digested PCR fragments were separated by electrophoresis in 2–3% agarose gel and detected with ethidium bromide staining. All PCRs were run in 10 µl volumes using 10–20 ng genomic DNA. Negative controls (water blanks) were included on each plate. BMI status was mixed blindly to control genotyping quality. Ninety-six randomly selected samples were genotyped twice for duplication accuracy. Sequencing analyses for 16 randomly selected samples with forward and reverse primers, respectively, were done using a Big-dye sequence kit (Applied Biosystem, ABI model 377 genetic analyzer, Foster City, USA).

### Statistical analysis and bioinformatics

One-way ANOVA was used to test differences in measured variables age, household income per person year, PAI and BMI. Qualitative variables and the allele frequencies and genotype distributions between cases and controls were compared by the Chi-square (χ^2^) test and a *p* value of 0.05 was defined to be statistically significant. Besides age and sex would affect the metabolism normality, low physical activity and household income were major determinants of obesity and overweight [16]–[17], multiple unconditional logistic regression (Enter method) was applied to evaluate and adjust for covariates, including age, sex, PAI and household income. The probability for entry is 0.05 and that for removal is 0.1. Statistical analyses as above were performed with Statistical Product and Service Solutions 13.0 (SPSS; SPSS Inc, Chicago, USA).

Hardy-Weinberg equilibrium (HWE) was assessed by Fisher's exact χ^2^ test using the program HWE in the control groups[18]. Haplo.score with R software (http://cran.r-project.org/) as outlined by Schaid et al. was used to test the associations of statistically inferred Haplotype with obesity and that models an individual's phenotype as a function of each inferred haplotype, weighted with their estimated probability, to account for haplotype ambiguity [19]. The Haplo.glm approach was performed to obtain the odds ratios (ORs) of risk Haplotype as well [20]. Both Haplo.score and Haplo.glm were implemented in Haplo.stats package, a suite of R routines for the analysis of indirectly measured Haplotype.

## Results

### Single marker association analyses

We conducted a genetic association analysis for 5 TagSNPs in the AC3 gene. Genotype distributions and allele frequencies of all studied SNPs in the population of Chinese Han adults were followed in Hardy-Weinberg equilibrium (HWE). Minor allele frequencies (MAFs) of the studied polymorphisms were shown in Table 2. Single marker association analysis with a dominant model indicated that SNP rs753529 was significantly associated with BMI in obese subjects (P = 0.026, OR = 0.780, 95% CI = 0.627–0.972). The frequencies of minor allele G in the groups of lean, overweight and obese subjects were gradually decreased from 20.6%, 9.7% to 5.2% but it didn't reached statistical significance (P value equal to 0.818 for overweight and 0.067 for obesity respectively).

Furthermore, logistic regression model was used to evaluate the effects of covariates. The association of SNP rs753529 with obesity still remained significant (P = 0.022, OR = 0.775, 95% CI = 0.623–0.963) after adjusted for covariates age, sex, PAI and household income among all single marker association analyses for obesity and overweight. Hosmer and Lemeshow Test of Goodness-of-Fit showed that Chi-square was 11.114 and P value was 0.195, and sex and PAI showed statistical significance in the model, ORs (95%CI) were 1.649 (1.293–2.103) and 0.984 (0.977–0.991) respectively.

Replication genotyping study of SNP rs753529 in children subjects was done, and comparison analyses between the groups of children with BMI = P_20–50_ and BMI>P_80_ were followed. Although the G allele frequency was lower in children subjects with BMP>P_80_ than in the subjects with BMI P_20–50_, no significant association of this polymorphism with BMI in children subjects was found (21.6% vs. 16.7%, P = 0.836).

Genetic association of SNP rs753529 with BMI in adults and children was summarized in table 3. No other studied SNPs were found to be associated with obesity in this Chinese Han population (see table S1).

**Table 3:** Genetic association of SNP rs753529 with BMI in adult and children subjects.

BMI	Genotypes				Alleles		
			OR(95% CI)	P-value	Major/minor	OR (95% CI)	P-value
Adult subjects	AA	AG+GG					
18.5–23.9	602	714+174	Reference		1918/1062	Reference	
24.0–27.9	274	307+96	0.978 (0.812–1.179)	0.818	855/499	1.054 (0.922–1.205)	0.440
≥28	192	176+45	0.775 (0.623–0.963)	0.022	560/266	0.858 (0.728–1.011)	0.067
Children subjects	AA	AG+GG					
P_20–50_	170	217+68	Reference		557/353	Reference	
>P_80_	138	174+49	0.969 (0.723–1.300)	0.836	450/272	1.054 (0.922–1.205)	0.644

Genotypic and allelic associations were tested, respectively, with dominant and additive models. All tests were adjusted for sex, age, PAI, household income and/or diet expanses.

### Haplotype association analyses

There were two LD blocks predicted by Haploview software in the studied Chinese Han population. We thus performed a multiple marker analysis with haplotypes (at least 5% frequency). The haplotypes in the first LD block were constructed by three SNPs, including rs1127568, rs7604576 and rs753529. Four common haplotypes were observed and the haplotype G-G-G was found to be significantly associated with obesity (P = 0.029, OR = 0.778, 95% CI = 0.621–0.975, Table 4). The effect was similar to the single SNP of rs753529 (OR = 0.775, OR95%CI = 0.623–0.963) and that indicated rs753529 lead independent genetic effect out of the first block.

**Table 4:** Common haplotypes constructed with SNPs rs1127568, rs7604576 and rs753529.

Haplotype	All subjects(n = 2580)	Lean subjects(n = 1490)	Obese subjects(n = 413)	OR (95%CI)	P-value
G-A-A	0.601	0.595	0.633	Reference	
G-G-G	0.167	0.168	0.142	0.778 (0.621–0.975)	0.029
A-G-G	0.124	0.127	0.127	0.909 (0.716–1.154)	0.433
G-A-G	0.059	0.061	0.050	0.784 (0.548–1.122)	0.183

Common haplotypes had >5% frequencies. All tests were adjusted for sex, age, PAI and household income.

Similar analyses with common haplotypes in the second LD block, which were constructed by SNPs rs1968482 and rs11676272, were performed but no significant association with BMI was found (see Table S2).

## Discussion

In the present study, we have conducted a genetic association study of the AC3 gene in a Chinese Han population. Data indicate that SNP rs753529 is associated with decreased risk of obesity in adults but not in children. The software of Power and Sample Size Calculation (Dupont WD, Plummer WD: http://biostat.mc.vanderbilt.edu/twiki/bin/view/Main/PowerSampleSize) was used to calculate the power. At the 5% significance level, we had 84.3% power to detect a dominant allele of rs753529 with MAF equal to 0.356 in control (n = 1490) and OR equal to 1.4 or 0.72 for obesity (n = 413) which has relative less sample than overweight (n = 677). Out of candidate SNPs, rs1127568 has lower MAF 0.13 in control and the power is 76.5% for OR equal to 1.5 or 0.67 for obesity.

Obesity is a heterogeneous disorder and genetic defects in different ethnic populations may be influenced by different genetic backgrounds and environmental factors. Nordman et al. have recently reported a genetic association study of the AC3 gene in a Swedish population and demonstrated that SNPs rs2033655 and rs1968482 in the AC3 gene had significant low minor allele frequencies in lean subjects compared with type 2 diabetes patients. These two polymorphisms are found to be associated significantly with obese subjects and obese type 2 diabetes patients (BMI≥30 kg/m^2^) but not significantly with non-obese type 2 diabetes patients (BMI≤26 or <30 kg/m^2^) [10]. These two SNPs have been included in the present study. Most likely due to the ethnic difference, SNP rs2033655 represents a low allele frequency in Chinese population and another closely linked SNP rs11676272 in Sweden is tested in this study, and neither the two SNPs nor their haplotype have significant association with obesity in the present study. Similarly, several genetic association studies of the adiponectin (ADIPOQ) gene in type 2 diabetes and obesity have been reported. The synonymous and intronic polymorphisms in exon 2 and intron 2 in the ADIPOQ gene are found to be associated with BMI in Japanese, Korean, Chinese and Caucasian populations, while the gene promoter polymorphisms are associated with BMI in French and Swedish populations [21]–[28].

Evidence from the genetic study suggests the interesting possibility that AC3 may play an important role in the regulation of body weight. Therefore, Wang et al. have generated a mouse model of AC3 deficiency to test this hypothesis. The AC3^−/−^ mice after birth are about half the size compared to wild type littermates, but achieve similar size and weight as wild control mice after two months. With the age of more than two moths, AC3^−/−^ mice become obese [11]. In the present study, we have found that the AC3 genetic polymorphisms are associated with obesity in adults and not in children. The data are consistent with the recent genetic and functional studies [10], [11]. The P-value of the association between SNP 753529 and BMI in children is 0.836, which is unlikely type 2 error caused by smaller sample size of children subjects in comparison with the adults.

Very recently, two genome wide association studies have demonstrated that the AC5 genetic polymorphisms are associated with type 2 diabetes in term of fasting glucose homeostasis [29], [30]. Interestingly, AC5 is another isoform of AC family are found to implicate. Both AC3 and AC5 are membrane-associated enzymes and activated/regulated by Gsα via forskolin, Ca^2+^/calmodulin dependent protein kinase [9]. Moreover, obesity is often associated with type 2 diabetes, and these two complex diseases may partially share the common issue in pathogenesis [31], [32]. At the present stage, however, we have limited knowledge regarding the role of AC3 and AC5 in patho-physiology of obesity and type 2 diabetes. Further biological investigation will be extremely of interest to understand of the impact of these two genes on obesity and/or type 2 diabetes.

In conclusion, the present study provides evidence that the AC3 genetic polymorphisms are associated with decreased risk of obesity in adults in a Chinese Han population. Taking together the recent and present genetic studies as well as evidence from the mouse model of AC3 deficiency, we suggest that the AC3 genetic effect on BMI may be interacted with the factors related to ageing and environment.

## Supporting Information

Table S1 Association analysis of AC3 gene with overweight and obesity.
(0.06 MB DOC)

Table S2 Haplotype frequencies of AC3 gene and association analysis with overweight and obesity.
(0.05 MB DOC)
