# Supplementary material for: Evaluation of the Association between the AC3 Genetic Polymorphisms and Obesity in a Chinese Han Population
Source: PLoS One. 2010 Nov 4;5(11):e13851. doi: 10.1371/journal.pone.0013851 (PMC2973974; doi:10.1371/journal.pone.0013851)
Supplement: Table S1 — Association analysis of AC3 gene with overweight and obesity. (0.06 MB DOC) [file pone.0013851.s001.doc]

Table S1 Association analysis of AC3 gene with overweight and obesity

| SNP | BMI group | Genotype (Dominant) | | | | |  | Allele | | |
| --- | --- | --- | --- | --- | --- | --- | --- | --- | --- | --- |
|  |  | WT | Ht+ MT |  | OR（95％CI）^a^ | P^a^ |  | Major/Minor | OR(95％CI)^b^ | P^b^ |
| rs11676272 |  | CC | CT+ TT |  |  |  |  |  |  |  |
|  | 18.5-23.9 | 483 | 753+253 |  | Reference |  |  | 1719/1259 | Reference |  |
|  | 24-27.9 | 208 | 350+118 |  | 1.073(0.880-1.307) | 0.487 |  | 766/586 | 1.045(0.917- 1.189) | 0.511 |
|  | ≥28 | 146 | 204+63 |  | 0.846(0.674-1.063) | 0.151 |  | 496/330 | 0.908(0.776-1.063) | 0.231 |
| rs1968482 |  | AA | AG+ GG |  |  |  |  |  |  |  |
|  | 18.5-23.9 | 571 | 694+225 |  | Reference |  |  | 1836/1144 | Reference |  |
|  | 24-27.9 | 238 | 319+120 |  | 1.130(0.934-1.368) | 0.209 |  | 795/559 | 1.128(0.990-1.286) | 0.070 |
|  | ≥28 | 169 | 190+54 |  | 0.870(0.697-1.085) | 0.216 |  | 528/298 | 0.906(0.772-1.063) | 0.226 |
| rs753529 |  | AA | AG+GG |  |  |  |  |  |  |  |
|  | 18.5-23.9 | 602 | 714+174 |  | Reference |  |  | 1918/1062 | Reference |  |
|  | 24-27.9 | 274 | 307+96 |  | 0.978(0.812-1.179) | 0.818 |  | 855/499 | 1.054(0.922-1.205) | 0.440 |
|  | ≥28 | 192 | 176+45 |  | 0.775(0.623-0.963) | 0.022 |  | 560/266 | 0.858(0.728-1.011) | 0.067 |
| rs7604576 |  | AA | AG+GG |  |  |  |  |  |  |  |
|  | 18.5-23.9 | 651 | 688+151 |  | Reference |  |  | 1990/990 | Reference |  |
|  | 24-27.9 | 285 | 310+82 |  | 1.040(0.864-1.252) | 0.675 |  | 880/474 | 1.083(0.946-1.239) | 0.249 |
|  | ≥28 | 195 | 177+41 |  | 0.827(0.665-1.029) | 0.089 |  | 567/259 | 0.918(0.778-1.084) | 0.312 |
| rs1127568 |  | GG | GA+AA |  |  |  |  |  |  |  |
|  | 18.5-23.9 | 1123 | 344+22 |  | Reference |  |  | 2590/388 | Reference |  |
|  | 24-27.9 | 518 | 142+16 |  | 0.921(0.743-1.143) | 0.455 |  | 1178/174 | 0.986(0.814-1.194) | 0.885 |
|  | ≥28 | 310 | 99+4 |  | 0.971(0.755-1.248) | 0.819 |  | 719/107 | 0.993(0.790-1.250) | 0.955 |

a Adjusted for gender, age, PAI and household income.

b OR estimated by Chi-square test

WT wide type, Ht heterozygote, MT mutant type
