# Supplementary material for: Evaluation of the Association between the AC3 Genetic Polymorphisms and Obesity in a Chinese Han Population
Source: PLoS One. 2010 Nov 4;5(11):e13851. doi: 10.1371/journal.pone.0013851 (PMC2973974; doi:10.1371/journal.pone.0013851)
Supplement: Table S2 — Haplotype frequencies of AC3 gene and association analysis with overweight and obesity. (0.05 MB DOC) [file pone.0013851.s002.doc]

Table S2 Haplotype frequencies of AC3 gene and association analysis with overweight and obesity

a Haplotype were listed with frequency over 1 percent.

| Haplotype ^a^ | | All (n=2580) | Controls  (n=1490) | Obesity | | |  | Overweight | | |
| --- | --- | --- | --- | --- | --- | --- | --- | --- | --- | --- |
|  |  |  |  | Frequency (n=413) | OR(95%CI) ^e^ | P value^e^ |  | Frequency  (n=677) | OR(95%CI)^e^ | P value^e^ |
| Block 1 ^b^ | |  |  |  |  |  |  |  |  |  |
| Hap1^d^ | G-A-A | 0.601 | 0.595 | 0.633 | reference |  |  | 0.587 | reference |  |
| Hap2 | G-G-G | 0.167 | 0.168 | 0.142 | 0.778(0.621-0.975) | 0.029 |  | 0.185 | 1.100(0.922-1.313) | 0.290 |
| Hap3 | A-G-G | 0.124 | 0.127 | 0.127 | 0.909(0.716-1.154) | 0.433 |  | 0.124 | 1.007(0.820-1.236) | 0.947 |
| Hap4 | G-A-G | 0.059 | 0.061 | 0.050 | 0.784(0.548-1.122) | 0.183 |  | 0.058 | 0.902(0.672-1.211) | 0.491 |
| Hap5 | G-G-A | 0.043 | 0.043 | 0.044 | 0.954(0.647-1.408) | 0.814 |  | 0.042 | 0.991(0.703-1.397) | 0.958 |
| Block 2^c^ | |  |  |  |  |  |  |  |  |  |
| Hap1^d^ | A-C | 0.542 | 0.536 | 0.573 | reference |  |  | 0.525 | reference |  |
| Hap2 | G-T | 0.352 | 0.352 | 0.334 | 0.882(0.745-1.045) | 0.146 |  | 0.372 | 1.090(0.947-1.256) | 0.230 |
| Hap3 | A-T | 0.070 | 0.074 | 0.066 | 0.809(0.590-1.109) | 0.189 |  | 0.062 | 0.856(0.651-1.125) | 0.265 |
| Hap4 | G-C | 0.036 | 0.037 | 0.027 | 0.704(0.439-1.129) | 0.145 |  | 0.041 | 1.179(0.829-1.678) | 0.360 |

b Loci are arranged in the order rs1127568- rs7604576-rs753529.

c Loci are arranged in the order rs1968482-rs11676272.

d Hap1 was chosen to be the reference.

e Adjusted for gender, age, PAI and household income.
